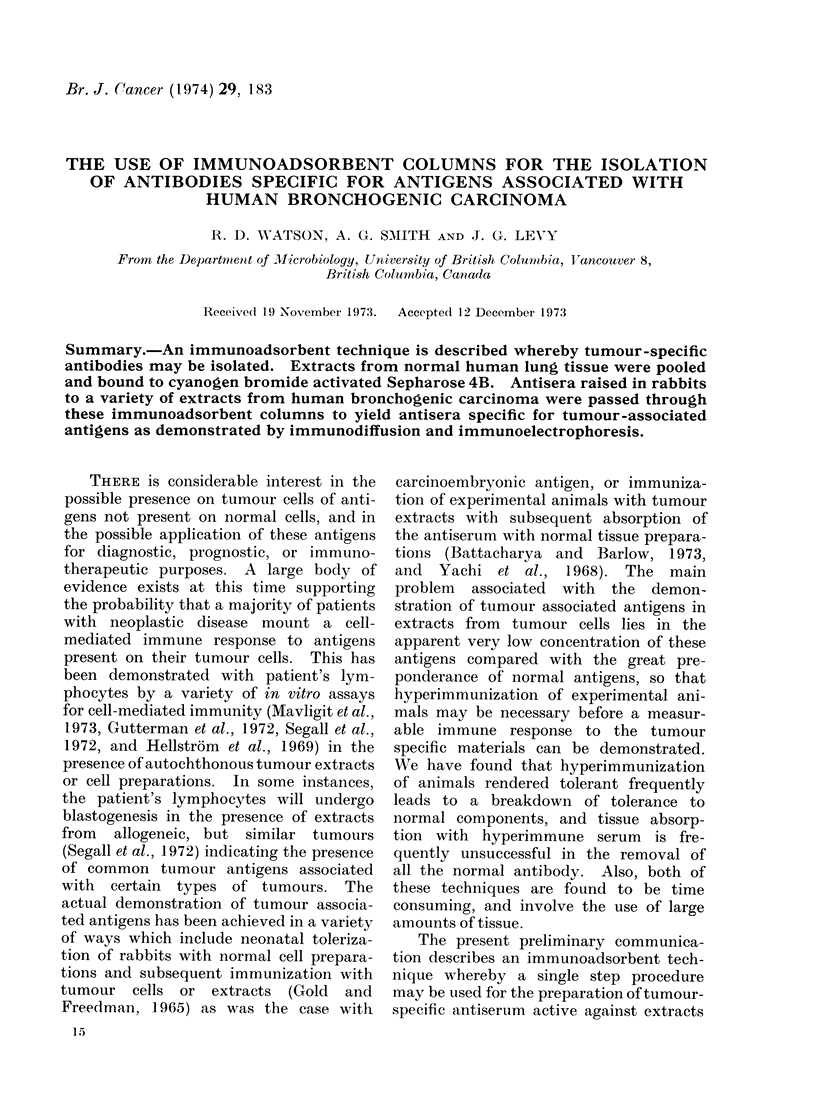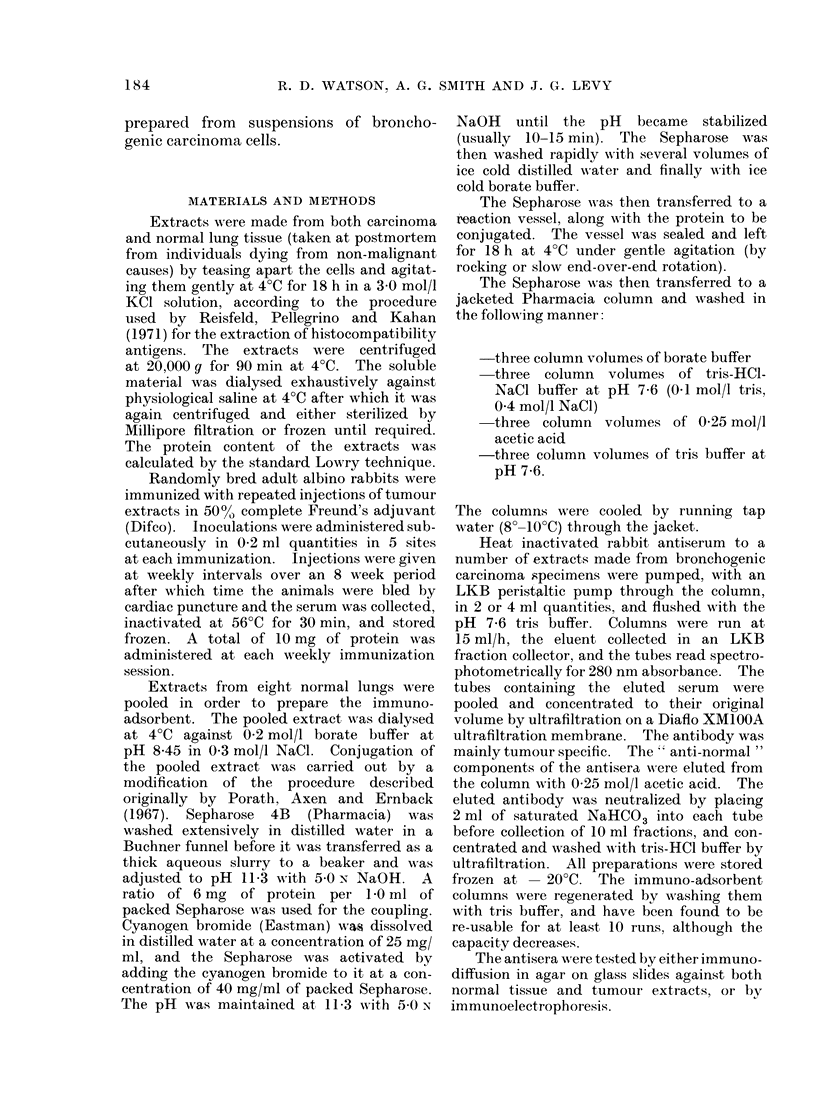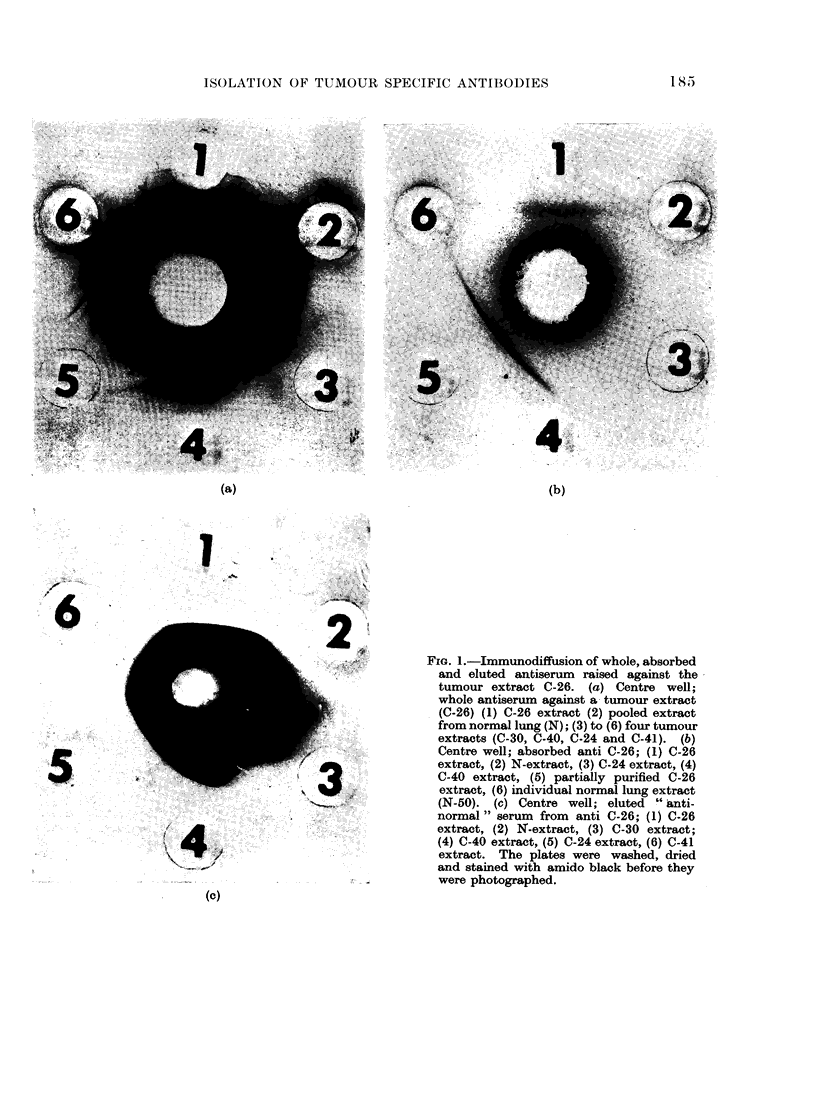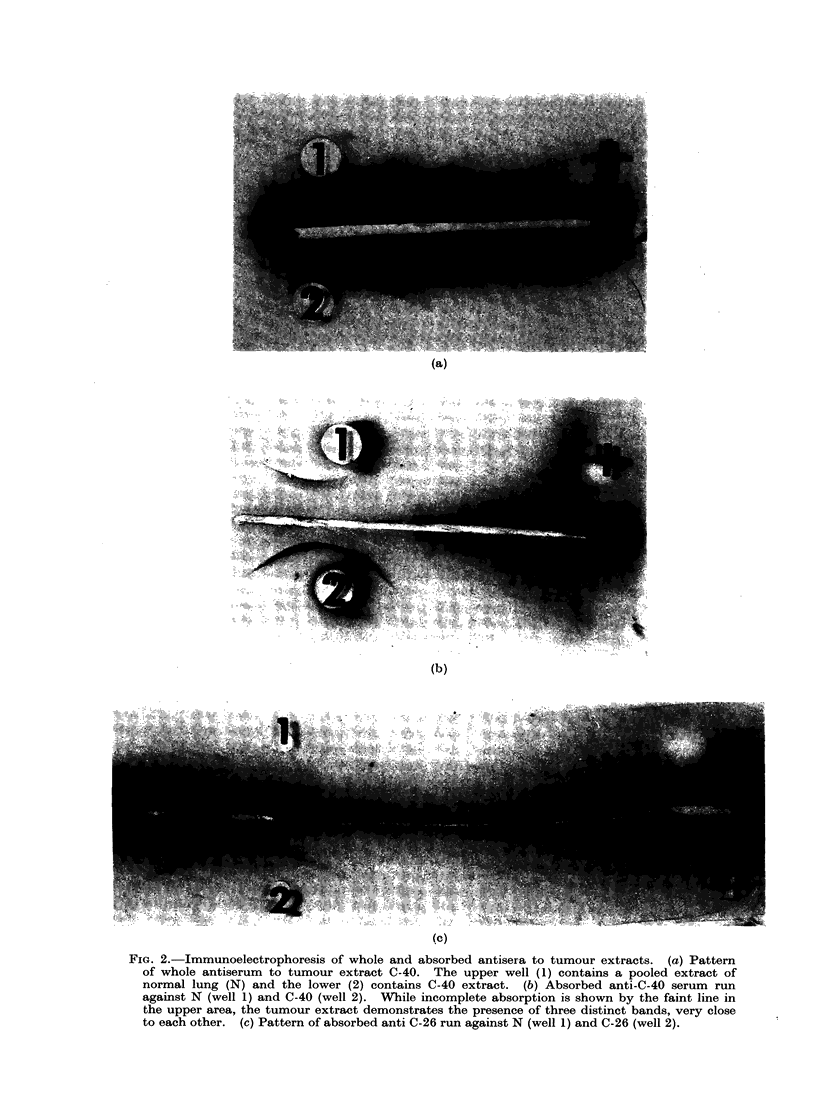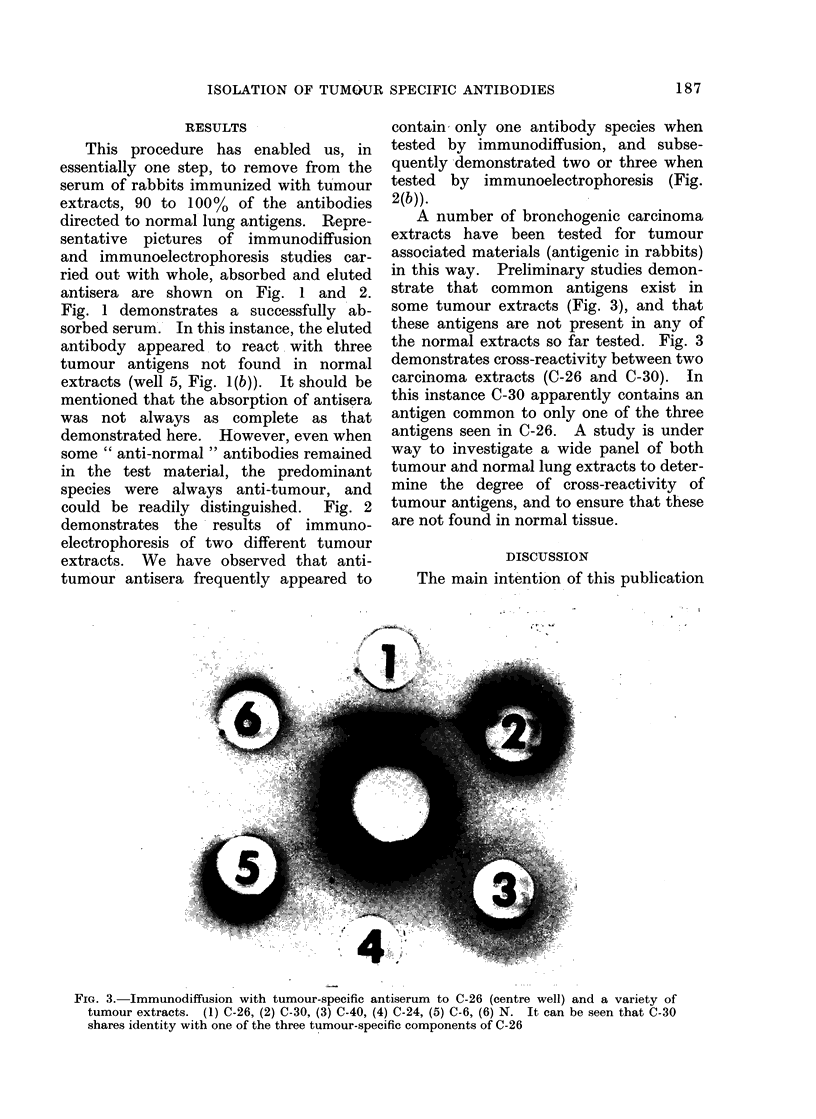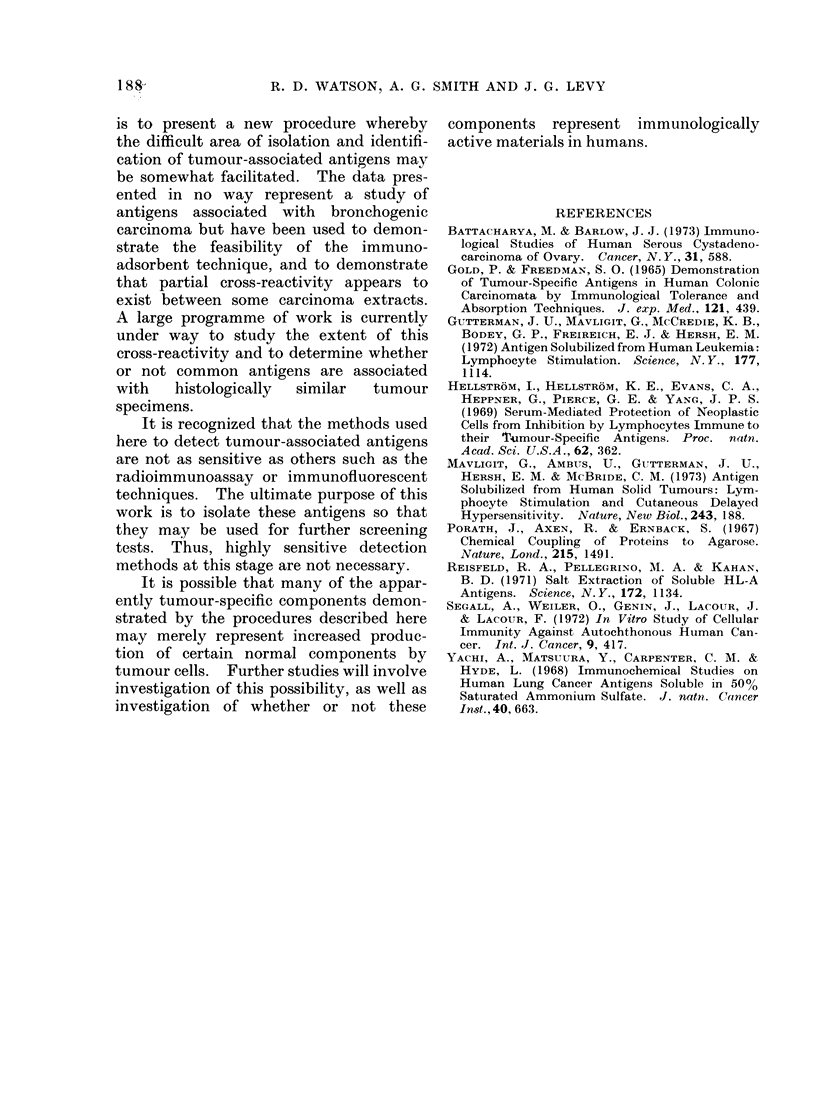# The Use of Immunoadsorbent Columns for the Isolation of Antibodies Specific for Antigens Associated with Human Bronchogenic Carcinoma

**DOI:** 10.1038/bjc.1974.57

**Published:** 1974-03

**Authors:** R. D. Watson, A. G. Smith, J. G. Levy

## Abstract

**Images:**


Br. J. Cancer (1974) 29, 183

### THE USE OF IMMUNOADSORBENT COLUMNS FOR THE ISOLATION OF ANTIBODIES SPECIFIC FOR ANTIGENS ASSOCIATED WITH HUMAN BRONCHOGENIC CARCINOMA

R. D. WATSON, A. G. SMITH AND J. G. LEVY

*From the Department of Microbiology, University of British Columbia, Vancouver 8, British Columbia, Canada*

Received 19 November 1973. Accepted 12 December 1973

**Summary.—An immunoadsorbent technique is described whereby tumour-specific antibodies may be isolated. Extracts from normal human lung tissue were pooled and bound to cyanogen bromide activated Sepharose 4B. Antisera raised in rabbits to a variety of extracts from human bronchogenic carcinoma were passed through these immunoadsorbent columns to yield antisera specific for tumour-associated antigens as demonstrated by immunodiffusion and immunoelectrophoresis.**

THERE is considerable interest in the possible presence on tumour cells of antigens not present on normal cells, and in the possible application of these antigens for diagnostic, prognostic, or immunotherapeutic purposes. A large body of evidence exists at this time supporting the probability that a majority of patients with neoplastic disease mount a cell-mediated immune response to antigens present on their tumour cells. This has been demonstrated with patient's lymphocytes by a variety of *in vitro* assays for cell-mediated immunity (Mavligit *et al.*, 1973, Gutterman *et al.*, 1972, Segall *et al.*, 1972, and Hellström *et al.*, 1969) in the presence of autochthonous tumour extracts or cell preparations. In some instances, the patient's lymphocytes will undergo blastogenesis in the presence of extracts from allogeneic, but similar tumours (Segall *et al.*, 1972) indicating the presence of common tumour antigens associated with certain types of tumours. The actual demonstration of tumour associated antigens has been achieved in a variety of ways which include neonatal tolerization of rabbits with normal cell preparations and subsequent immunization with tumour cells or extracts (Gold and Freedman, 1965) as was the case with carcinoembryonic antigen, or immunization of experimental animals with tumour extracts with subsequent absorption of the antiserum with normal tissue preparations (Battacharya and Barlow, 1973, and Yachi *et al.*, 1968). The main problem associated with the demonstration of tumour associated antigens in extracts from tumour cells lies in the apparent very low concentration of these antigens compared with the great preponderance of normal antigens, so that hyperimmunization of experimental animals may be necessary before a measurable immune response to the tumour specific materials can be demonstrated. We have found that hyperimmunization of animals rendered tolerant frequently leads to a breakdown of tolerance to normal components, and tissue absorption with hyperimmune serum is frequently unsuccessful in the removal of all the normal antibody. Also, both of these techniques are found to be time consuming, and involve the use of large amounts of tissue.

The present preliminary communication describes an immunoadsorbent technique whereby a single step procedure may be used for the preparation of tumour-specific antiserum active against extracts prepared from suspensions of bronchogenic carcinoma cells.

### MATERIALS AND METHODS

Extracts were made from both carcinoma and normal lung tissue (taken at postmortem from individuals dying from non-malignant causes) by teasing apart the cells and agitating them gently at 4°C for 18 h in a 3·0 mol/l KCl solution, according to the procedure used by Reisfeld, Pellegrino and Kahan (1971) for the extraction of histocompatibility antigens. The extracts were centrifuged at 20,000 *g* for 90 min at 4°C. The soluble material was dialysed exhaustively against physiological saline at 4°C after which it was again centrifuged and either sterilized by Millipore filtration or frozen until required. The protein content of the extracts was calculated by the standard Lowry technique.

Randomly bred adult albino rabbits were immunized with repeated injections of tumour extracts in 50% complete Freund's adjuvant (Difco). Inoculations were administered subcutaneously in 0·2 ml quantities in 5 sites at each immunization. Injections were given at weekly intervals over an 8 week period after which time the animals were bled by cardiac puncture and the serum was collected, inactivated at 56°C for 30 min, and stored frozen. A total of 10 mg of protein was administered at each weekly immunization session.

Extracts from eight normal lungs were pooled in order to prepare the immunoadsorbent. The pooled extract was dialysed at 4°C against 0·2 mol/l borate buffer at pH 8·45 in 0·3 mol/l NaCl. Conjugation of the pooled extract was carried out by a modification of the procedure described originally by Porath, Axen and Ernback (1967). Sepharose 4B (Pharmacia) was washed extensively in distilled water in a Buchner funnel before it was transferred as a thick aqueous slurry to a beaker and was adjusted to pH 11·3 with 5·0 N NaOH. A ratio of 6 mg of protein per 1·0 ml of packed Sepharose was used for the coupling. Cyanogen bromide (Eastman) was dissolved in distilled water at a concentration of 25 mg/ml, and the Sepharose was activated by adding the cyanogen bromide to it at a concentration of 40 mg/ml of packed Sepharose. The pH was maintained at 11·3 with 5·0 N NaOH until the pH became stabilized (usually 10–15 min). The Sepharose was then washed rapidly with several volumes of ice cold distilled water and finally with ice cold borate buffer.

The Sepharose was then transferred to a reaction vessel, along with the protein to be conjugated. The vessel was sealed and left for 18 h at 4°C under gentle agitation (by rocking or slow end-over-end rotation).

The Sepharose was then transferred to a jacketed Pharmacia column and washed in the following manner:

—three column volumes of borate buffer
—three column volumes of tris-HCl-NaCl buffer at pH 7·6 (0·1 mol/l tris, 0·4 mol/l NaCl)
—three column volumes of 0·25 mol/l acetic acid
—three column volumes of tris buffer at pH 7·6.

The columns were cooled by running tap water (8°–10°C) through the jacket.

Heat inactivated rabbit antiserum to a number of extracts made from bronchogenic carcinoma specimens were pumped, with an LKB peristaltic pump through the column, in 2 or 4 ml quantities, and flushed with the pH 7·6 tris buffer. Columns were run at 15 ml/h, the eluent collected in an LKB fraction collector, and the tubes read spectrophotometrically for 280 nm absorbance. The tubes containing the eluted serum were pooled and concentrated to their original volume by ultrafiltration on a Diaflo XM100A ultrafiltration membrane. The antibody was mainly tumour specific. The " anti-normal " components of the antisera were eluted from the column with 0·25 mol/l acetic acid. The eluted antibody was neutralized by placing 2 ml of saturated $NaHCO_3$ into each tube before collection of 10 ml fractions, and concentrated and washed with tris-HCl buffer by ultrafiltration. All preparations were stored frozen at − 20°C. The immuno-adsorbent columns were regenerated by washing them with tris buffer, and have been found to be re-usable for at least 10 runs, although the capacity decreases.

The antisera were tested by either immunodiffusion in agar on glass slides against both normal tissue and tumour extracts, or by immunoelectrophoresis.

(a)

(b)

(c)

FIG. 1.—Immunodiffusion of whole, absorbed and eluted antiserum raised against the tumour extract C-26. (*a*) Centre well; whole antiserum against a tumour extract (C-26) (1) C-26 extract (2) pooled extract from normal lung (N); (3) to (6) four tumour extracts (C-30, C-40, C-24 and C-41). (*b*) Centre well; absorbed anti C-26; (1) C-26 extract, (2) N-extract, (3) C-24 extract, (4) C-40 extract, (5) partially purified C-26 extract, (6) individual normal lung extract (N-50). (*c*) Centre well; eluted " anti-normal " serum from anti C-26; (1) C-26 extract, (2) N-extract, (3) C-30 extract; (4) C-40 extract, (5) C-24 extract, (6) C-41 extract. The plates were washed, dried and stained with amido black before they were photographed.

FIG. 2.—Immunoelectrophoresis of whole and absorbed antisera to tumour extracts. (*a*) Pattern of whole antiserum to tumour extract C-40. The upper well (1) contains a pooled extract of normal lung (N) and the lower (2) contains C-40 extract. (*b*) Absorbed anti-C-40 serum run against N (well 1) and C-40 (well 2). While incomplete absorption is shown by the faint line in the upper area, the tumour extract demonstrates the presence of three distinct bands, very close to each other. (*c*) Pattern of absorbed anti C-26 run against N (well 1) and C-26 (well 2).

### RESULTS

This procedure has enabled us, in essentially one step, to remove from the serum of rabbits immunized with tumour extracts, 90 to 100% of the antibodies directed to normal lung antigens. Representative pictures of immunodiffusion and immunoelectrophoresis studies carried out with whole, absorbed and eluted antisera are shown on Fig. 1 and 2. Fig. 1 demonstrates a successfully absorbed serum. In this instance, the eluted antibody appeared to react with three tumour antigens not found in normal extracts (well 5, Fig. 1(*b*)). It should be mentioned that the absorption of antisera was not always as complete as that demonstrated here. However, even when some " anti-normal " antibodies remained in the test material, the predominant species were always anti-tumour, and could be readily distinguished. Fig. 2 demonstrates the results of immunoelectrophoresis of two different tumour extracts. We have observed that anti-tumour antisera frequently appeared to contain only one antibody species when tested by immunodiffusion, and subsequently demonstrated two or three when tested by immunoelectrophoresis (Fig. 2(*b*)).

A number of bronchogenic carcinoma extracts have been tested for tumour associated materials (antigenic in rabbits) in this way. Preliminary studies demonstrate that common antigens exist in some tumour extracts (Fig. 3), and that these antigens are not present in any of the normal extracts so far tested. Fig. 3 demonstrates cross-reactivity between two carcinoma extracts (C-26 and C-30). In this instance C-30 apparently contains an antigen common to only one of the three antigens seen in C-26. A study is under way to investigate a wide panel of both tumour and normal lung extracts to determine the degree of cross-reactivity of tumour antigens, and to ensure that these are not found in normal tissue.

### DISCUSSION

The main intention of this publication

FIG. 3.—Immunodiffusion with tumour-specific antiserum to C-26 (centre well) and a variety of tumour extracts. (1) C-26, (2) C-30, (3) C-40, (4) C-24, (5) C-6, (6) N. It can be seen that C-30 shares identity with one of the three tumour-specific components of C-26

is to present a new procedure whereby the difficult area of isolation and identification of tumour-associated antigens may be somewhat facilitated. The data presented in no way represent a study of antigens associated with bronchogenic carcinoma but have been used to demonstrate the feasibility of the immunoadsorbent technique, and to demonstrate that partial cross-reactivity appears to exist between some carcinoma extracts. A large programme of work is currently under way to study the extent of this cross-reactivity and to determine whether or not common antigens are associated with histologically similar tumour specimens.

It is recognized that the methods used here to detect tumour-associated antigens are not as sensitive as others such as the radioimmunoassay or immunofluorescent techniques. The ultimate purpose of this work is to isolate these antigens so that they may be used for further screening tests. Thus, highly sensitive detection methods at this stage are not necessary.

It is possible that many of the apparently tumour-specific components demonstrated by the procedures described here may merely represent increased production of certain normal components by tumour cells. Further studies will involve investigation of this possibility, as well as investigation of whether or not these components represent immunologically active materials in humans.

### REFERENCES

BATTACHARYA, M. & BARLOW, J. J. (1973) Immunological Studies of Human Serous Cystadenocarcinoma of Ovary. *Cancer, N.Y.*, **31**, 588.

GOLD, P. & FREEDMAN, S. O. (1965) Demonstration of Tumour-Specific Antigens in Human Colonic Carcinomata by Immunological Tolerance and Absorption Techniques. *J. exp. Med.*, **121**, 439.

GUTTERMAN, J. U., MAVLIGIT, G., MCCREDIE, K. B., BODEY, G. P., FREIREICH, E. J. & HERSH, E. M. (1972) Antigen Solubilized from Human Leukemia: Lymphocyte Stimulation. *Science, N.Y.*, **177**, 1114.

HELLSTRÖM, I., HELLSTRÖM, K. E., EVANS, C. A., HEPPNER, G., PIERCE, G. E. & YANG, J. P. S. (1969) Serum-Mediated Protection of Neoplastic Cells from Inhibition by Lymphocytes Immune to their Tumour-Specific Antigens. *Proc. natn. Acad. Sci. U.S.A.*, **62**, 362.

MAVLIGIT, G., AMBUS, U., GUTTERMAN, J. U., HERSH, E. M. & MCBRIDE, C. M. (1973) Antigen Solubilized from Human Solid Tumours: Lymphocyte Stimulation and Cutaneous Delayed Hypersensitivity. *Nature, New Biol.*, **243**, 188.

PORATH, J., AXEN, R. & ERNBACK, S. (1967) Chemical Coupling of Proteins to Agarose. *Nature, Lond.*, **215**, 1491.

REISFELD, R. A., PELLEGRINO, M. A. & KAHAN, B. D. (1971) Salt Extraction of Soluble HL-A Antigens. *Science, N.Y.*, **172**, 1134.

SEGALL, A., WEILER, O., GENIN, J., LACOUR, J. & LACOUR, F. (1972) *In Vitro* Study of Cellular Immunity Against Autochthonous Human Cancer. *Int. J. Cancer*, **9**, 417.

YACHI, A., MATSUURA, Y., CARPENTER, C. M. & HYDE, L. (1968) Immunochemical Studies on Human Lung Cancer Antigens Soluble in 50% Saturated Ammonium Sulfate. *J. natn. Cancer Inst.*, **40**, 663.